# Manual of Detection of Poisons

**Published:** 1858-03

**Authors:** 


ART. VII.—*A Manual of the Detection of Poisons by Medico-Chemical Analysis.* By Dr. FR. JUL. OTTO, Professor of Chemistry in Caroline College, Brunswick. Translated from the German, by WILLIAM ELDERHORST, M.D., Professor of Chemistry in the Rensselaer Polytechnic Institute, Troy, New York. 12mo. pp. 178. H. Baillière: New York, 1857.

THE Manual of Professor Otto is one pre-eminently adapted for the purposes for which it is intended, and will, doubtless, meet with a proper appreciation from those taking an interest in toxicological investigations. Though not designed as a complete treatise on the subject, it is, nevertheless, sufficiently full for all practical purposes. The rationale of many of the processes is given with some degree of minuteness, and all can be readily understood by any one having a slight acquaintance with the principles of chemistry.

Nearly half the book is taken up with the consideration of the means for the detection of arsenic. The circumstances which may lead to error in the employment of Marsh's test are well pointed out, and the method of Fresenius and Babo—undoubtedly the best we at present possess for the discovery of this poison—is given in detail.

The remaining portion of the work is devoted to the consideration of the methods for the detection of antimony, mercury, copper, lead, tin, zinc, hydrocyanic acid, oxalic acid, phosphorus, alcohol, chloroform, and the poisonous alkaloids, and concludes with a chapter on the examination of blood-stains.

The translator has performed his work in a creditable manner, and has, besides other additions, furnished the whole of the chapter on oxalic acid.

We repeat that the Manual is an excellent guide to toxicological researches. It fills a void in our medical literature which has long existed, and we wish for it an extended circulation.

